# Fracture of the Os Hyodies

**Published:** 1857-08

**Authors:** Frank H. Hamilton


### BUFFALO MEDICAL JOURNAL

AND

### MONTHLY REVIEW.

**VOL. 13.** AUGUST, 1857. **NO. 3.**

### ORIGINAL COMMUNICATIONS.

ART. I.—FRACTURE OF THE OS HYOIDES. (*Syn. Fracture of the Tongue-bone. Fractura ossis hyoidei.*) By FRANK H. HAMILTON, M. D.

M. Orfila has reported the case of a man, aged 62 years, who had been hanged, and whose os hyoides was broken through its body on its right side.* M. Cazauvièilh has also seen a fracture of this bone in two persons who had been hanged: in one of which the fracture was probably in the body of the bone, and in the other through one of its cornua.†

Lalesque published in the "Journal Hebdomadaire," for March, 1833, a case which occurred in a marine, 67 years old, "who, in a quarrel had his throat violently clenched by the hand of a vigorous adversary. At the moment there was very acute pain, and the sensation of a solid body breaking. The pain was aggravated by every effort to speak, to swallow, or to move the tongue, and when this organ was pushed backward. Deglutition was impossible, the patient could not articulate distinctly; and he was unable to

* Traité de Méd. legale, 3d ed. tom. 2, p. 423. Also Traité des Frac. et des Lux., par L. F. Malgaigne, vol. i, p. 405.

† Cazauvièilh, du Suicide, etc., p. 221. Also Malg. op. cit., p. 405.

open his mouth without exciting a great deal of pain. He placed his hand upon the anterior and superior part of his neck to point out the seat of the injury. This part was slightly swollen, and presented on each side small ecchymosis, one above, more decided, immediately under the left angle of the lower jaw. The large cornua of the os hyoides was very distinctly to the right side, and it could be felt on the left deeply seated by pressing with the fingers; in following it in front toward the body of the bone, a very sensible inequality near the point of junction of these two parts could be perceived. By putting the finger within the mouth, the same projections and cavities inverted could be felt, and even the points of the bone which had pierced the mucous membrane, &c., were evident. Having bled the patient, and placed a plug between his teeth to keep the mouth open, the broken branch was brought by the finger back to the surface of the body of the bone, and easily reduced. The position of the head inclined a little back; rest, absolute silence, diet and some saturnine fomentations, composed the after-treatment. To avoid a new dislocation, by the efforts of swallowing, the æsophagus tube of Desault was introduced, to conduct the drinks and liquid aliments into the stomach; this sound was allowed to remain until the twenty-fifth day, at this time the patient could swallow without pain, and began to take a little more solid nourishment, and at the end of two months the cure was complete. By placing a finger within his mouth, a slight nodosity could be felt in the place where in the recent fracture the splintered points were perceptible.*

Dieffenbach has also recorded a fracture of the great, right horn, produced in the same manner, by grasping the throat between the thumb and fingers, which occurred in a young girl only 19 years old. Very slight pressure upon the side of the bone was sufficient to move the fragment inward, and to produce a crepitus, but it immediately resumed its place when the pressure was removed. There being, therefore, no displacement, the cure was effected in a short time without resort to any remedies except tisans and antiphlogistics. She was not even forbidden to speak.†

Auberge saw a similar case, in a person 55 years old, occasioned by grasping the throat. The fracture was in the great horn of the right side, and the displacement was so complete that crepitus could not be felt, and the mucous membrane of the pharynx was penetrated by the broken bone.‡

---

* Amer. Jour. Med. Sci., vol. xiii, p. 250.

† Medic. Vereinszeitung für Preussen, 1833, No. 3. Gazette Med., 1834, p. 187. Also Malg. op. cit., p. 405.

‡ Revue Méd., July, 1835. Also Malg. op. cit., p. 405.

The following example is reported by Dr. Wood, of Cincinnati, Ohio, as having come under his observation in the year 1855:

"Through the kindness of our friend Dr. P. G. Fore, of this city, we were invited to examine a case of fracture of the os hyoides, that had occurred about one week before we saw it, in one of his patients. The patient was a female, about thirty years of age, who had fallen down the cellar steps, striking the prominent part of the larynx and hyoid bone against a projecting brick, severely injuring the larynx as well as fracturing the bone.

"The fracture was on the left side, and near the junction of the great horn with the body of the bone. Crepitation was distinctly felt on pressing the bone between the thumb and finger; or when the patient would swallow; though, at this time, the severe symptoms that followed the accident, and continued for several days, had somewhat subsided.

"Immediately after the accident, there was profuse bleeding from the fauces, and she experienced great difficulty and pain in the act of swallowing, and the power of speech was almost entirely lost. On attempting to depress or protrude the tongue, she felt distressing symptoms of suffocation. Considerable inflammation and swelling of the throat and larynx ensued, and continued in some degree up to the time of our visit.

"To-day (about four weeks since the accident) Dr. F. informs us that the patient has so far recovered as to be able to converse, though the voice is somewhat impaired. She is yet unable to swallow solid food, and is wholly sustained by fluids."*

Marcinkovsky saw a woman in whom both the lower jaw and the left horn of the os hyoides were broken by a fall from her carriage against a wall. She died in about twenty-four hours with suffocation.†

Dr. Gründer reports the following:

"A laborer, æt. 63, fell from a wagon on his face, and discharged a large quantity of blood by the mouth. He found he could not swallow, and when seen twelve hours afterward, complained of severe pain in the neck and nape, with inability to turn his head, though no injury of the vertebræ could be detected. His voice was hoarse and difficult. On attempting to drink, the fluid was rejected with violent coughing, the patient declaring he felt it as if entering the air passages. An examination of the fauces led to no explanation of this condition. The epiglottis did not, however, appear to completely

---

* Western Lancet. Also N. Y. Jour· Med., vol. xv, p. 152.

† Medic. Vereinszeitung, für Preussen, 1833, No. 15. Gazette Médicale, 1833, p. 354. Also Malg. op. cit., p. 405.

close the larynx, or to be in its exact position. The tongue was movable in all directions, and pressing it down with a spatula caused no inconvenience. The hyoid seemed to possess its continuity. No crepitation or abnormal movability could be perceived, and no pain at the root of the tongue occurred on attempting to swallow. After repeated examinations, the case was concluded to be one in which the functions of the nervus vagus had undergone great disturbance, or the muscles of the larynx had become torn or paralyzed. Medicine and food were administered by means of an elastic tube. The patient had a good appetite and slept well; the pain of the neck was lost, and its motion recovered; a hectic cough, from which he had long suffered, alone remaining. After continuing, however, to go on thus well for six days, the cough increased; the appetite failed; strength was lost; the voice was scarcely audible; and in five more days the patient died exhausted. At the autopsy a fracture of the os hyoides was found. One of the large cornua was broken, and had become firmly imbedded between the epiglottis and rima glottidis, inducing the raised position of the epiglottis, loss of voice and difficulty in swallowing. The fracture was probably produced by muscular action, a cause first assigned in a case occurring to Ollivier d'Angers."* I think it more probable, however, that this fracture was the result of a direct blow, than of muscular action.

In the case referred to, however, as having been reported by Ollivier, there can be no doubt that the fracture was due to muscular action alone.

A woman, fifty-six years old, made a misstep and fell backward, and at the same moment that her head was thrown violently back, she felt distinctly a sensation as if a solid body had broken in the upper part of her neck, and upon her left side. An examination showed that she had fractured the great, left horn of the os hyoides. Inflammation and suppuration followed, and finally, after about three months, the posterior fragment made its way out in a condition of necrosis, and the fistula promptly healed, but there remained for many years a sense of uneasiness about these parts when she swallowed, sometimes amounting to pain.†

*Etiology.* Of the ten cases which I have found upon record, three were produced by hanging; three by grasping the throat between the thumb and fingers; three by direct blows, or by falls upon the front of the neck; and one by muscular action alone.

---

* Schmidt's Jahrbuch., vol. 68. Also Amer. Jour. Med. Sci., vol. 49, p. 253, Jan., 1852.

† Malg. op. cit., p. 405.

The observation of Mr. South that fracture of the bone "is almost invariably found"* in persons executed by hanging, is probably incorrect, since although a large proportion of these subjects are submitted to dissection both in this and in other countries, yet I know of but these three examples which have been published.

*Pathology, Symptomatology and Diagnosis.* The body of the bone seems to have been broken in all of those cases which resulted from hanging; while in all of the other examples the fracture has occurred in one of the great horns, or at the junction of the horns with the body. Generally the displacement inward of one of the fragments has been so complete as that crepitus could not be detected. It was present, however, in the examples mentioned by Dieffenbach and Wood. In two instances the mucous membrane has been penetrated, and in one the fragment was projected between the epiglottis and rima glottidis.

The accident has been characterized by a sudden sensation as if a bone had broken; in a few instances, by profuse bleeding from the fauces; by difficulty in opening the mouth; by impossibility of deglutition, and by loss of voice in others; with great pain in moving the tongue, the pain being especially at its root; in one instance the tongue was perceptibly drawn to one side. There is also usually more or less swelling and soreness about the neck, with ecchymosis; and at a later period, cough, expectoration, hoarseness, &c. The circumstances which, however, indicate certainly the nature of the accident, are preternatural mobility of the fragments, with or without crepitus, and the angular, inward projection, which may in most cases be distinctly felt in a careful examination of the pharynx.

In the case related by Grüner, the only symptoms were a loss of voice, difficulty of deglutition, and a sensation when the attempt was made, as if the fluids passed into his windpipe; with also an imperfect closure of the epiglottis upon the rima glottidis. No preternatural mobility or irregularity in the fragments could be detected, nor was there crepitus, and it was concluded that the bone was not broken, yet the autopsy showed that the fragment was imbedded deeply between the epiglottis and the rima glottidis.

*Prognosis.* It is only in view of its complications that this accident can be regarded as serious: where the severity of the injury has been such as to fracture the lower jaw at the same time, as in the case related by Marcin-

---

* Note to Chelins' Surgery, Amer. Ed., vol. i, p. 581.

kovsky, or such as to bury the fragment deep in the tissues about the rima glottidis as in the case mentioned by Grüner, a favorable termination could scarcely have been expected; and these are the only cases yet published in which the death was in any way connected with the fracture. One-half of the whole number have died, but of these, three died by hanging, and the remaining two from the causes named. Of the three in whom the accident resulted from a direct blow, only the patient of Dr. Fore, of Cincinnati, has survived; while of the three whose fractures resulted from lateral pressure upon the cornua, all recovered; so also, did the patient in whom the fracture was produced by muscular action.

*Treatment.* No doubt when the fragments are displaced an attempt ought to be made to replace them by introducing one finger into the mouth, whilst with the opposite hand the fragments are supported from without. Lalesque found this a matter of some difficulty, but Auberge experienced no difficulty at all. I suspect, however, that the amount of difficulty will very much depend upon the degree of displacement, and the consequent laceration of the soft tissues about the bone. But however this may be, it must be altogether another thing to be able to keep in exact apposition the broken ends of a bone whose diameter is so inconsiderable, and upon which it is quite impossible to apply any apparatus or dressings to retain the fragments in place. Lalesque threw the head of his patient slightly back, with the view of making "permanent extension" upon the fragments through the action of the muscles and ligaments attached to the bone, and he recommends this position as that which is best calculated to preserve the coaptation. Malgaigne, on the contrary, without having himself seen any example of this fracture, believes that the position of flexion of the neck, with entire relaxation of the muscles would be most suitable.

In all cases it will be proper to enjoin silence, and to adopt suitable measures to combat inflammation: such as general or topical bleeding, fomentations, moistening the mouth with cool water, or permitting small pieces of ice to rest in the mouth until dissolved, without allowing the fluid to be swallowed. In other examples, no doubt the patient may be permitted to swallow.

### Fracture of the Cartilages of the Larynx. (*Syn. Fractura Laryngis.*)

*Thyroid Cartilage.* The examples of fracture of the larynx which may be found upon record, are also very few. M. Ladoz examined the larynx of a man who had been assassinated, and upon whose neck he found a handkerchief bound so tightly as to leave, after its removal, a deep furrow; but the neck showed also distinct marks produced by the fingers and thumb. There was a fracture of the thyroid cartilage which extended obliquely downward and outward through its right wing. The whole of the larynx was very much ossified, although the subject was only thirty-seven years old.*

In 1823, M. Ollivier communicated to the Academy of Medicine a case in which, this cartilage being broken, the patient died of suffocation.†

M. Marjolin says, "two women at the hospital being engaged in a quarrel, one of them seized her antagonist by the throat, and gripped her so strong that she broke the thyroid cartilage from its upper to its lower margin. You will imagine that it was not very difficult to determine the existence of a fracture, and that no retentive apparatus was demanded. Silence, regimen, a small bleeding, and the cure was accomplished."‡

These are the only cases of fracture of the cartilages of the larynx of which we have any precise account, in which the thyroid cartilage was alone involved.

*Thyroid and Cricoid Cartilages.* Plenck saw a fracture of both the thyroid and cricoid cartilages produced by falling upon the rim of a pail.‖ Morgagni also says that he had seen fractures of the larynx; and Remer mentions a fracture of the larynx found in a person who had been hanged;§ but in neither case is it said in which cartilage the fracture occurred, or whether it had not occurred in both.

I am able, however, to furnish from my own observation another example of fracture of both cartilages:

John Calkins, of Collins, Erie Co, N. Y., æt. 41, is supposed to have been kicked by a young horse on the 10th of Nov., 1856. He was alone in the stables when the accident occurred, and being stunned by the blow, he was unable himself to give any account of the manner in which the injury was received. When found, he was sitting upright, but unable to articulate, except in a whisper. Drs. Barber and Davis, of Colden, saw him about two

---

* Gazette Médicale, 1838, p. 698. Also Traité des Fractures et des Luxations, par L. F. Malgaigne, vol. i, p. 409.

† Archives Générales de Médicine, tome ii, p. 307. Also Malg., op. cit., p. 409.

‡ Marjolin, Cours de Patholog. Chir., p. 396. Also Malg., op. cit., p. 409.

‖ Malg., op. cit., p. 409.

§ Morgagni, de Sedibus, etc., Epist. 19, num. 13, 14 et 16; Remer, Annales d'hygiene, tome 4, p. 171. Also Malg., op. cit., p. 408.

hours after. His countenance was anxious; his pulse feeble; extremities cold; and he was breathing with great difficulty. A small quantity of blood was issuing from his fauces. His upper lip was cut and a few of his teeth dislocated: the wound appearing as if inflicted by one of the corks of the horse's shoes. There was no other wound; but over the left wing of the thyroid cartilage there was a slight discoloration, pressure upon which produced intense pain and suffocation, and disclosed the fact that the thyroid prominence was depressed very much and broken. Cold lotions were directed to be applied, and as the thirst was excessive, but deglutition impossible, he was permitted to hold pieces of ice in his mouth. This plan, with but slight modifications, such as the substitution of warm fomentations to the neck for the cold lotions, was continued until the following evening, when, at the request of the attending physician, Dr. Barber, I was called to see him. The symptoms remained nearly the same as at first. He was unable to speak audibly, or perform the act of deglutition; his breathing was difficult and at times threatened suffocation. The lateness of the hour, with other circumstances, determined me to defer surgical interference until morning. At day break of the 12th I made the operation of laryngotomy, and introduced a large double canula into the crico-thyroidean space. This operation was rendered difficult by the great amount of swelling about the neck, due both to emphysema, and bloody with serous infiltrations. The breathing immediately became easy, and gradually the appearance of asphyxia disappeared from his face; but after about six or seven hours, he began perceptibly to fail in strength, and died at 3 o'clock, P. M., of the following day, apparently from exhaustion rather than from suffocation: having survived the accident about seventy-two hours, and the operation about thirty-four hours.

The autopsy disclosed a comminuted fracture of the thyroid cartilage, with a simple fracture of the cricoid. The thyroid was broken almost perpendicularly through its centre; the line of fracture being irregular, and inclining slightly to the left side. The left inferior horn was broken off about three lines from its articulation with the cricoid cartilage. The right ala was broken also in a line nearly vertical, but irregular, at a point about six lines from its posterior margin. The pomum adami was depressed to the level of the cricoid cartilage, and the left ala, being completely detached, was thrown inward and upward several lines. Underneath the perichondrium, especially upon the inner side, there was pretty extensive bloody infiltration. Ossification of the cartillages had commenced at several points, but it had made but little progress. The central fracture of the thyroid was through carti lage alone. The fracture of the right ala was through cartilage until it reached a bony belt comprising the two inferior lines of its course. The left lower horn was ossified, and the fracture was through this bony structure. The fracture through the cricoid cartilage commenced close upon the margin of a bony plate, but in its whole course it traversed only cartilage. It was on the left side. There was also an incomplete fracture on the right ala of the thyroid cartilage, commencing in the line of the principle fracture and extending obliquely downward about three lines, until it was arrested by the bony plate which constituted the lower margin of this wing.

A ragged, lacerated wound in the back of the larynx, above the cricoid cartilages, communicated directly with the æsophagus.

*Cricoid Cartilage.* Both Valsalva and Cazauvieilh have each met with a single example of this fracture, without fracture of the thyroid; and Weiss has found the cricoid cartilage broken into numerous fragments, and at the same time separated from the trachea.*

*Etiology.* As a predisposing cause, advanced age, with its usual concomitant, partial or complete ossification of the cartilages, has been thought to occupy a prominent place. The number of recorded cases is, however, too small to establish its actual value. In the case reported by Plenck, the cartilages were already very much ossified, although the subject was only 37 years old. Morgagni observed that in his experience it had occurred always in advanced life. In my own case, however, the cartilages were only slightly ossified, the patient being 41 years old; nor did the lines of the several fractures indicate a preference for the bony plates; but it seems to me that they rather avoided them, and in the case of the incomplete fracture, the bone appeared to have arrested the fracture. In fact, a few experiments have satisfied me that the adult laryngeal cartilages are quite as brittle as bone, and, consequently, that ossification in no way increases their liability to fracture.

The immediate causes have been direct blows, as from falling upon the edge of a pail, a kick from a horse; or pressure, as in hanging, or by grasping the larynx strongly between the thumb and fingers.

*Symptomatology and Diagnosis.* The signs of this accident are such as usually attend any severe injury of this organ, whether accompanied with a

---

* Malg., op. cit., p. 408.

fracture or not: such as pain, swelling, difficult deglutition, embarrassed respiration, a loss of voice, with cough, and perhaps bloody expectoration, with emphysema, &c.

But none of these can be regarded as diagnostic; although, when taken in connection with the history of the accident, especially if a very severe and direct blow has been received, or more certainly still, when symptoms so grave and complicated have followed an attempt at strangulation by grasping the throat, they may be regarded as probable or presumptive evidences.

A positive diagnosis must depend upon the presence of a sensible displacement, or motion of the fragments, with crepitus.

In the case related by Plenck, death followed almost immediately, with convulsions, and without any outcry: indicating, probably, some severe lesion of the spinal marrow; while in M. Ollivier's patient suffocation ensued, at first intermittent, and finally permanent.

In my own case, suffocation was throughout a prominent symptom, with only such slight intervals of amelioration as might have been occasioned by the extrication of the blood or mucus from the larynx.

*Prognosis.* The prognosis ought to depend rather upon the complications and upon the gravity of the symptoms, than upon the simple decision of the question of fracture. A fracture produced by grasping the wings of the thyroid cartilage, and without any great contusion or laceration of the soft parts, might reasonably be expected to terminate favorably under judicious management; but when, on the contrary, the fracture is the result of great violence inflicted directly upon the front of the cartilages, producing severe contusion and laceration, and followed by great swelling, very difficult respiration, complete aphonia, impossibility of deglutition, &c., the prognosis cannot but be unfavorable—and indeed the woman spoken of by Marjolin, whose larynx was broken by grasping the neck, is the only one, so far as we know, whose recovery has been mentioned.

*Treatment.* In examples of simple, uncomplicated fracture, "silence, regimen and a small bleeding," may suffice; but in other cases, it may become necessary to introduce a tube into the stomach to supply the patient with food and drinks, since deglutition may be impossible. If also, suffocation is imminent, there may remain no alternative but a resort to tracheotomy, or to laryngotomy. I am not aware that this has ever been practised except by myself, yet its propriety, under certain conditions, is sufficiently manifest.

As to a "reduction" of the fragments, by manipulation, I believe it will be found generally, if not always, impracticable. Whatever displacement exists must be mostly inward, and we can have no means of forcing them again outward. Nor if once replaced, do I see any reason to suppose that they would not become immediately displaced.

Chelins has suggested the propriety in such cases, of cutting open the coverings of the larynx freely in the mesian line, and after stanching the bleeding, proceeding at once to divide the larynx itself in its whole length, and then replacing the broken cartilages.* The procedure has an aspect of severity, but I can well conceive of circumstances which would justify its adoption; not, however, so much for the purpose of replacing the cartilages, as for the purpose of arresting a fatal internal hæmorrhage, and of giving a free admission of air to the lungs. If this operation were to be practised, the wound ought to be left open for a sufficient length of time to allow of the subsidence of the inflammation, and then permitted to close with such precautions as experience teaches are usually necessary after the windpipe has been opened.

Active antiphlogistic measures, combined with fomentations to the neck, so far as these latter are found to be agreeable and practicable, are important measures, and not to be overlooked in the general plan of treatment.

My own patient, also, found small pieces of ice, permitted slowly to dissolve in the mouth, very grateful, but he preferred very much as an external application, the warm fomentations to the cold lotions.

* System of Surgery, Philadelphia Ed., vol. i, p. 581. 1847.

